# Using big data to understand bilingual performance in semantic fluency: Findings from the Canadian Longitudinal Study on Aging

**DOI:** 10.1371/journal.pone.0277660

**Published:** 2022-11-28

**Authors:** Vanessa Taler, Brendan Johns

**Affiliations:** 1 School of Psychology, University of Ottawa, Ottawa, Canada; 2 Bruyère Research Institute, Ottawa, Canada; 3 Department of Psychology, McGill University, Montreal, Canada; University of Padova, ITALY

## Abstract

**Objectives:**

This study aimed to characterize verbal fluency performance in monolinguals and bilinguals using data from the Canadian Longitudinal Study on Aging (CLSA).

**Methods:**

A large sample of adults aged 45–85 (n = 12,875) completed a one-minute animal fluency task in English. Participants were English-speaking monolinguals (n = 9,759), bilinguals who spoke English as their first language (L1 bilinguals, n = 1,836), and bilinguals who spoke English as their second language (L2 bilinguals, n = 1,280). Using a distributional modeling approach to quantify the semantic similarity of words, we examined the impact of word frequency and pairwise semantic similarity on performance on this task.

**Results:**

Overall, L1 bilinguals outperformed monolinguals on the verbal fluency task: they produced more items, and these items were of lower average frequency and semantic similarity. Monolinguals in turn outperformed L2 bilinguals on these measures. The results held across different age groups, educational, and income levels.

**Discussion:**

These results demonstrate an advantage for bilinguals compared to monolinguals on a category fluency task, when performed in the first language, indicating that, at least in the CLSA sample, bilinguals have superior semantic search capabilities in their first language compared to monolingual speakers of that language.

## Introduction

The prevalence of bilingualism worldwide is high and ever-increasing, with estimates that more than 50% of the world’s population is bilingual [1]. It is well-established that monolinguals outperform bilinguals in language tasks such as picture naming [2–4]. However, the findings with respect to performance on executive function tasks are less definitive. Some studies have reported a so-called “bilingual advantage” in tasks of executive functioning, including executive control, inhibition, and switching [e.g., 3, 5], while others have found no such effect [e.g., 6] [for a critique of this study, see 7], and others have found mixed results [e.g., 8].

One of the most commonly-used cognitive tasks in both research and clinical practice is verbal fluency. In this task, the individual is asked to produce as many words as possible conforming to a given criterion within a certain time period, typically one minute. The criterion may be semantic (e.g., animals) or orthographic (e.g., words starting with the letter F). Fluency tasks assess both executive function and language processing. A meta-analysis of fluency performance in people with focal cortical lesions [9] suggested differential contributions of executive and language function depending on the criterion: category (semantic) fluency appears to rely more heavily on left temporal lobe integrity, suggesting a greater reliance on semantic processing, while both letter (orthographic) and category fluency rely on frontal structures related to executive function. In an exploratory factor-analytic study, in contrast, Whiteside et al. [10] found that both category and letter fluency loaded onto a language factor rather than an executive factor, suggesting that language is the critical component for this task.

Fluency performance is typically assessed in terms of the total number of items produced. However, analysis of the individual items produced is also instructive. Influential early research in this area used a clustering-and-switching approach, which examines the subgrouping of items within a category (clusters, e.g., pets, farm animals) and switches between clusters [11]. This approach has been ground-breaking in terms of identifying changes in fluency performance in various disorders such as Alzheimer’s and Parkinson’s disease [12], Huntington’s disease [13], and traumatic brain injury [14].

More recently, novel computational approaches have been developed to reduce subjectivity in scoring and eliminate the need for binary classification of responses as either belonging to a cluster or not [15, 16]. Instead, these models quantify the path taken through semantic memory as the speaker responds. These approaches have been used to examine fluency performance in various populations, such as middle-aged and older adults [17], cognitively healthy older adults vs. people who go on to develop mild cognitive impairment [18], people with schizophrenia [19, 20], and bilinguals [21].

The key advancement that has led to these new models is the development of distributional models of semantics (e.g., the Latent Semantic Analysis (LSA) model of Landauer & Dumais [22]). Distributional models learn the meaning of words based on co-occurrence statistics across sizeable samples of natural language, and have proven to be essential in cognitive and language sciences [for recent reviews, see 23, 24]. A wide number of different distributional model types have been proposed [22, 25–28] but all are based upon the exploitation of co-occurrence patterns of words in context. The result of training a distributional model is an accurate representation of a word’s meaning within a multi-dimensional space (although there are some notable limitations in the models, such as a lack of grounded information being included in their representations; see [29]).

The representation that distributional models construct can then be used as an underlying representation to drive processing models of cognition [30], including verbal fluency [e.g., 15]. Specifically, the models allow for the pairwise similarity of words produced during a verbal fluency task to be computed, which can then be used to determine what types of information are being used to drive the memory search process (e.g., semantic similarity or word frequency). This allows for a quantitative determination of how different information sources are being used across different populations while performing the task.

Given the central role of fluency tasks in clinical assessment, as well as the ongoing debate regarding the impact of bilingualism on cognitive performance, it is critical to characterize the performance of bilinguals on this task. Several studies have found that bilinguals perform worse than monolinguals on category fluency tasks, even when they are conducted in the participant’s dominant language [3, 31–33, although see 34]. Differences in the items produced have also been reported. Sandoval et al. [35] found that bilinguals produced significantly lower-frequency words and a greater proportion of cognate responses (i.e., words with a similar form in the two languages) compared to monolinguals. Using a computational approach similar to the one employed here, Taler et al. [21] examined fluency performance in English monolinguals and English-French bilinguals, and found similar performance in English-language fluency across the two groups. In a condition with high executive demands (forced language switch, where participants were required to alternate between French and English on each item), bilinguals produced items of higher frequency, and semantic similarity between adjacent items was lower. We have also reported similar findings regarding the dynamical usage of word frequency and semantic similarity in older adults who went on to develop mild cognitive impairment compared to those who remained cognitively healthy [18], as well as middle-aged relative to older adults [17]. These findings suggest that when memory search becomes more difficult, people tend to switch to producing items that are higher in word frequency and that are closer together in semantic space due to limitations in the availability of low frequency words and long-distance semantic connections in participants facing complex task demands or cognitive decline.

In the present study, we use distributional modelling to examine category fluency performance in a large group of middle-aged and older monolinguals and bilinguals, drawn from the Canadian Longitudinal Study on Aging [CLSA; 36], an ongoing long-term study of more than 50,000 Canadians. We have previously reported on the effects of age on category fluency performance in this sample [17], finding that across all participants, the sequence of items produced in the category fluency task was best predicted by semantic neighborhood, and that as age increased, the items produced were of higher frequency and from denser semantic neighborhoods. Here we extend these analyses to examine the fluency performance of monolinguals and bilinguals across the aging spectrum. Production of higher-frequency items and words that are closer together in semantic space in bilinguals relative to monolinguals would indicate a bilingual *disadvantage* on verbal fluency, while an *advantage* would be reflected in production of items that are of lower frequency and more spread out in semantic space. Stronger effects of bilingualism in older adults [37] would be supported by an interaction between age and bilingualism. To control for language of administration, we subdivided bilinguals into first language (L1) English and second language (L2) English. All participants included here completed the fluency task in English.

## Methods

Data for the present study are from the CLSA, which includes a national stratified sample of >50,000 Canadians aged 45–85 at baseline. Detailed information on CLSA design and methodology are provided in Raina et al. [36]. The CLSA is a 20-year prospective cohort study that includes two participant groups: a Tracking Cohort, who provide data through telephone interviews (*n* > 20,000) and a Comprehensive Cohort who provide data through in-home interviews and assessment at a data collection site (*n* > 30,000). Ethical review of the CLSA protocol was conducted by the Ethical, Legal, and Social Issues Committee, falling under the jurisdiction of the Canadian Institutes of Health Research (CIHR), and research ethics board approval was then acquired from each research site. Participation in the CLSA cohort is voluntary, and all CLSA participants provided written informed consent. The present study uses baseline data from the Comprehensive Cohort and was approved by the research ethics boards at Bruyère Research Institute and University of Ottawa. All data used in the current analyses were fully anonymized. This research was completed in accordance with the Helsinki Declaration.

### Participants

Participants who are part of the Comprehensive Cohort and who completed the fluency task in English are included in the present study. Data are from the baseline wave of data collection. Exclusion criteria included a diagnosis of dementia or Alzheimer’s disease, a memory disorder, Parkinson’s disease, or brain cancer, and stroke or traumatic brain injury. The CLSA data encodes the number of languages in which a participant can maintain a conversation. Participants who reported only being able to conduct a conversation in English were categorized as monolingual (N = 9,759), while participants who reported being able to hold a conversation in English and one other language were categorized as bilinguals. Multilinguals were not included in the present analyses. The bilingual group was further split into those who reported that English was the language they first learned at home (L1 group; N = 1,836), and those who reported learning a different language first (L2 group; N = 1,280; total N = 12,875). The average age of the monolingual group was 63.21 (SD = 10.12), the average for the bilingual L1 group was 61.9 (SD = 10.02), and the average for the bilingual L2 group was 63.27 (SD = 10.653).

For detailed discussion of the CLSA cognitive assessment, please see Taler et al. [38]. Included in the cognitive battery is an animal fluency task, in which participants name as many animals as they can in one minute (for descriptive analyses of performance in this task by CLSA participants, see [39]).

### Model description and corpus

The BEAGLE model of distributional semantics was used here to analyze differences in the usage of semantic similarity across the different participant groups, following a line of research using this model to examine fluency performance [15, 17, 18, 40]. Given the success of this model to examine verbal fluency in previous studies, we chose this model to keep the results consistent with previous analyses. BEAGLE learns the meaning of words by learning both context and order information at the sentence level. Like most distributional models, BEAGLE stores the meaning of a word in a vector, with each word having its own unique representation.

BEAGLE uses two types of vectors–environmental vectors, **e**, and memory vectors, **m**. Environmental vectors are stable (i.e., they do not change across modeling training) and serve as unique identifiers for the occurrence of a word in a corpus. Each of the *i* unique words in the model’s vocabulary has a unique environmental vector, **e**_*i*_. Vectors in BEAGLE are Gaussian vectors with a mean of zero and a standard deviation of $\sqrt{1/n}$ where n is the dimensionality of the vectors (set at 1,024). The environmental vectors are used to build up the memory vectors, which represent the meaning of words through the “reading” of a text corpus.

The model reads one sentence at a time and updates its memory vectors accordingly. Each word has two types of memory vectors: *context* and *order*. When a word occurs in a sentence, its context memory vector is updated by summing all the other words that occurred in the sentence with that word. The result of this learning mechanism is that the context vectors for words that tend to occur in the same sentence types grow more similar across model training.

For the order memory vector, the goal is to learn simplified syntactic information about a word’s usage by encoding the n-grams (up to a certain size; the default size is up to n-grams of size 7, which is used here) in which a word occurs. N-grams are encoded using noncommunicative circular convolution [41] inspired by the operations utilized by the classic episodic memory model TODAM of [42] to bind environmental vectors into unique n-gram vectors. The n-gram representations are then summed into a word’s order memory vector, accumulating syntactic information across sentential experiences. The end result of this process is an order vector that encodes unique information about how a word is used in combination with other words, which has been shown to account for unique information in lexical semantics [e.g., 43–45].

Thus, in the BEAGLE model, the context vector contains pure co-occurrence information, while the order vector encodes simplified syntactic information. The representation used here is the combination of the context and order vector, obtained by summing both vectors into a single composite for each word. Even though the model is quite simple, it provides a powerful account for a wide variety of language phenomena [27, 46, 47]. The main measure that will be taken from the BEAGLE model is the cosine (normalized dot product) similarity between words, produced within a verbal fluency task. That is, average pairwise similarity of words produced will be the metric derived from the model. The formula to derive this value is given by:

$$pairwise\;similarity=\frac{\sum_{i=2}^{n}cosine(w_{i-1},w_{i})}{n}$$

Where *n* is the number of words produced, *w* is the vector representation of a word, and *i* goes through each word produced.

The corpus that was used to train the model was a corpus of fiction and non-fiction books containing more than 2 billion words. This corpus was previously used to examine age effects on fluency performance in CLSA [17], to explain lexical organization data [48], to explore the impact of demographics on language processing [49], and to assess gender bias in language usage [50]. The corpus was preprocessed to change multiword animal names into single words (e.g., “grizzly bear” into “grizzlybear”). Word frequency was also assessed from this corpus, by counting each time that a word occurred in the corpus.

### Analysis technique

To analyze the data, multivariate ANOVAs were employed. This technique was used because we felt it was the best way to isolate the main effects and visualize the data across the various sociodemographic factors. This technique allows for a clear determination of the impact of language group on the dependent variables (namely, number of items produced, average word frequency, and pairwise semantic similarity). Bonferroni post-hoc tests were employed in the follow-up analysis, due to the size of the dataset and the fact that this is a conservative post-hoc test. These analyses were conducted using the full dataset. Given the important effects of age, education, and socio-economic status on cognitive performance, we then conducted a series of ANOVAs where participants were stratified by age, years of education, and income (as a proxy for socio-economic status), in order to control for these potential confounds.

## Results

**Fig 1:**
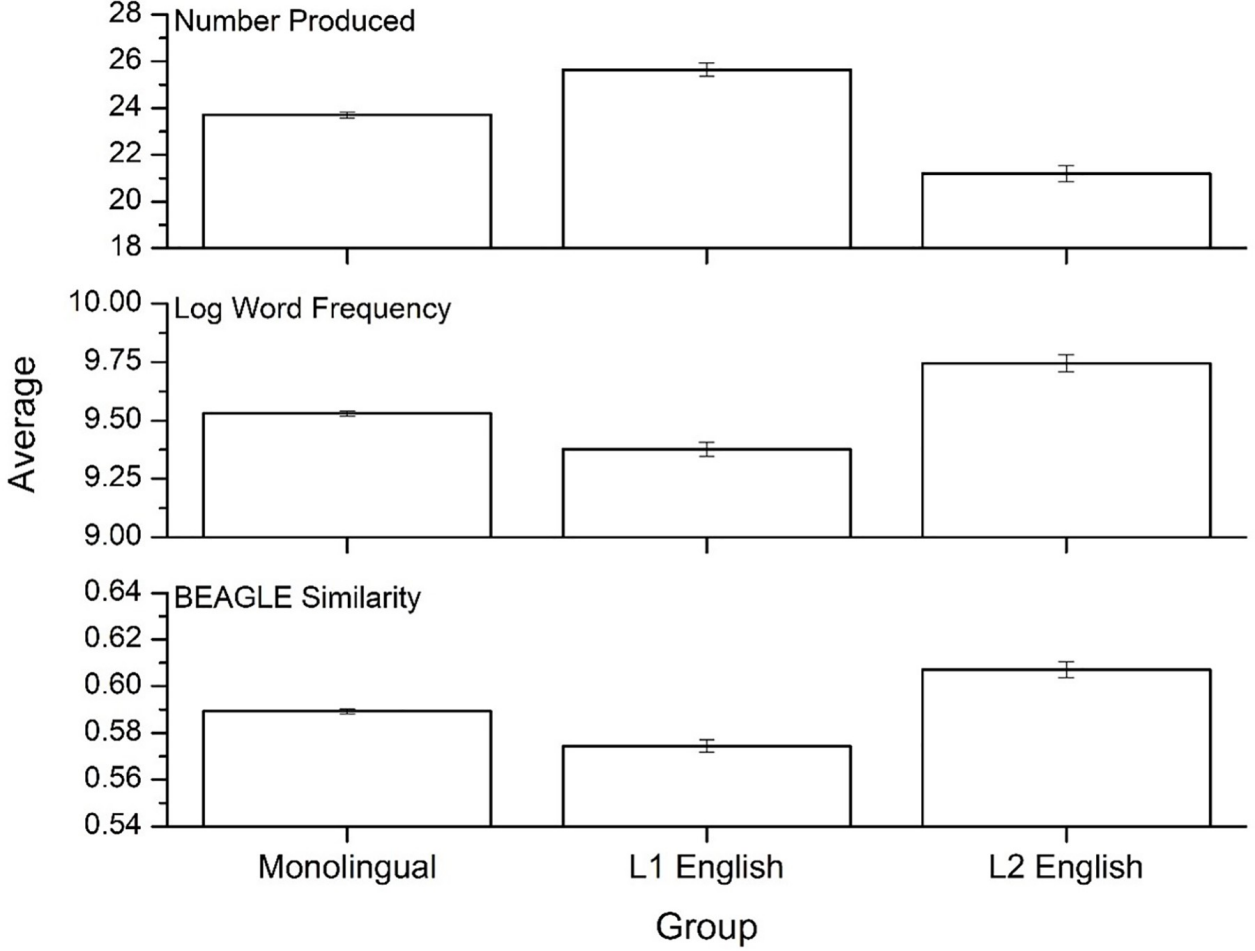
Means for the three psycholinguistic variables across the three language groups. Error bars represent 95% confidence intervals.

Fig 1 shows the means for the variables under question (total items, average log word frequency, average pairwise BEAGLE similarity) for the three language groups (monolinguals, English L1 bilinguals, and English L2 bilinguals). Group differences were analyzed using a multivariate ANOVA. For total items, a significant main effect of group was found [F(2, 13,776) = 186.31, p<0.001, η^2^ = .026]. A Bonferroni post-hoc test confirmed that L1 bilinguals produced significantly more items than monolinguals and L2 bilinguals, and monolinguals produced more items than L2 bilinguals (p<0.001 in all cases). Significant main effects of group was also found for word frequency [F(2, 13,776) = 156.96, p<0.001, η^2^ = .022] and semantic similarity [F(2, 13,776) = 119.35, p<0.001, η^2^ = .017]. Bonferroni post-hoc tests found that L2 bilinguals produced higher frequency items than monolinguals (p<0.001), who produced higher frequency items than L1 bilinguals (p<0.001). The semantic similarity metric showed identical trends, with L2 bilinguals having the highest semantic similarity, followed by monolinguals and then L1 bilinguals (p<0.001). In sum, L1 bilinguals produced more items in the animal fluency task compared to monolinguals and L2 English bilinguals, and these items were lower in both word frequency and semantic similarity.

We then considered three potentially relevant demographic characteristics: age, education, and income. For the age analyses, we assessed the correlation between age and the three variables of interest (total items produced, word frequency, and semantic similarity; results are provided in Table 1). For number of items, the monolingual and L1 English groups showed a stronger negative correlation than the L2 English group, suggesting that there is a greater age-related decline in output in monolinguals and L1 English participants compared to L2 English participants. However, given that the L2 English group produced significantly fewer items overall, this may be a floor effect. Across all three groups, correlations with age and lexical variables (word frequency and semantic similarity) were positive, suggesting that older people tend to produce items that are higher in word frequency and closer together in semantic space. The weakest correlations were seen in the L2 English group, suggesting that age-related changes in the use of lexical statistics are lowest in this group.

**Table 1:** Correlations between age and the three psycholinguistic variables for the difference language groups.

	# Produced	Word frequency	BEAGLE sim
Monolingual	-.307	.201	.201
L1 English	-.322	.157	.166
L2 English	-.207	.124	.121

**Note.** All correlations significant at the p<0.001 level.

**Fig 2:**
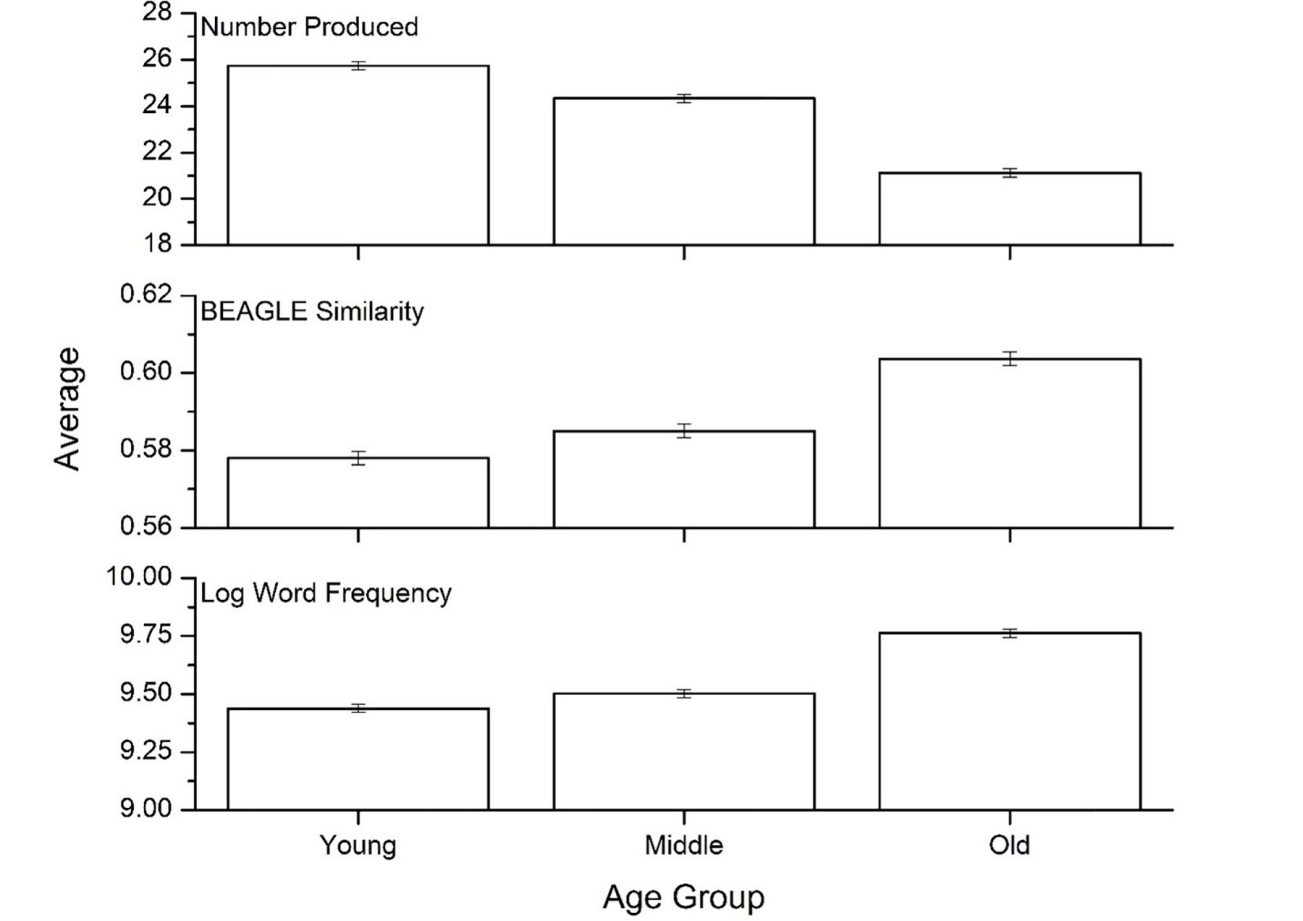
Means for the three psycholinguistic variables across the three age groups. Error bars represent 95% confidence intervals.

To ensure that the same trends displayed in Fig 1 hold across age groups, the data were then split into three groups: 1) younger (<58), 2) middle-aged (58 to 67), and 3) older (68+). Groups were of roughly equal size (monolingual: younger N = 3428, middle-aged N = 3544, older N = 3498; L1 English: younger N = 736, middle-aged N = 636, older N = 556; L2 English: younger N = 476, middle-aged N = 400, older N = 503). Fig 2 shows performance by age, collapsed across the three language groups. There was a significant effect of age on total items [F(2, 13,776) = 636.49, p<0.001, η^2^ = .085], word frequency [F(2, 13,776) = 230.23, p<0.001, η^2^ = .032], and semantic similarity [F(2, 13,776) = 224.89, p<0.001, η^2^ = .032]. Bonferroni post-hoc tests indicated that the younger group produced more items than the middle-aged and older groups, and word frequency and semantic similarity were lower (p<0.001). Similarly, the middle-aged group produced more items that were of lower word frequency and semantic similarity, compared to the older group.

**Fig 3:**
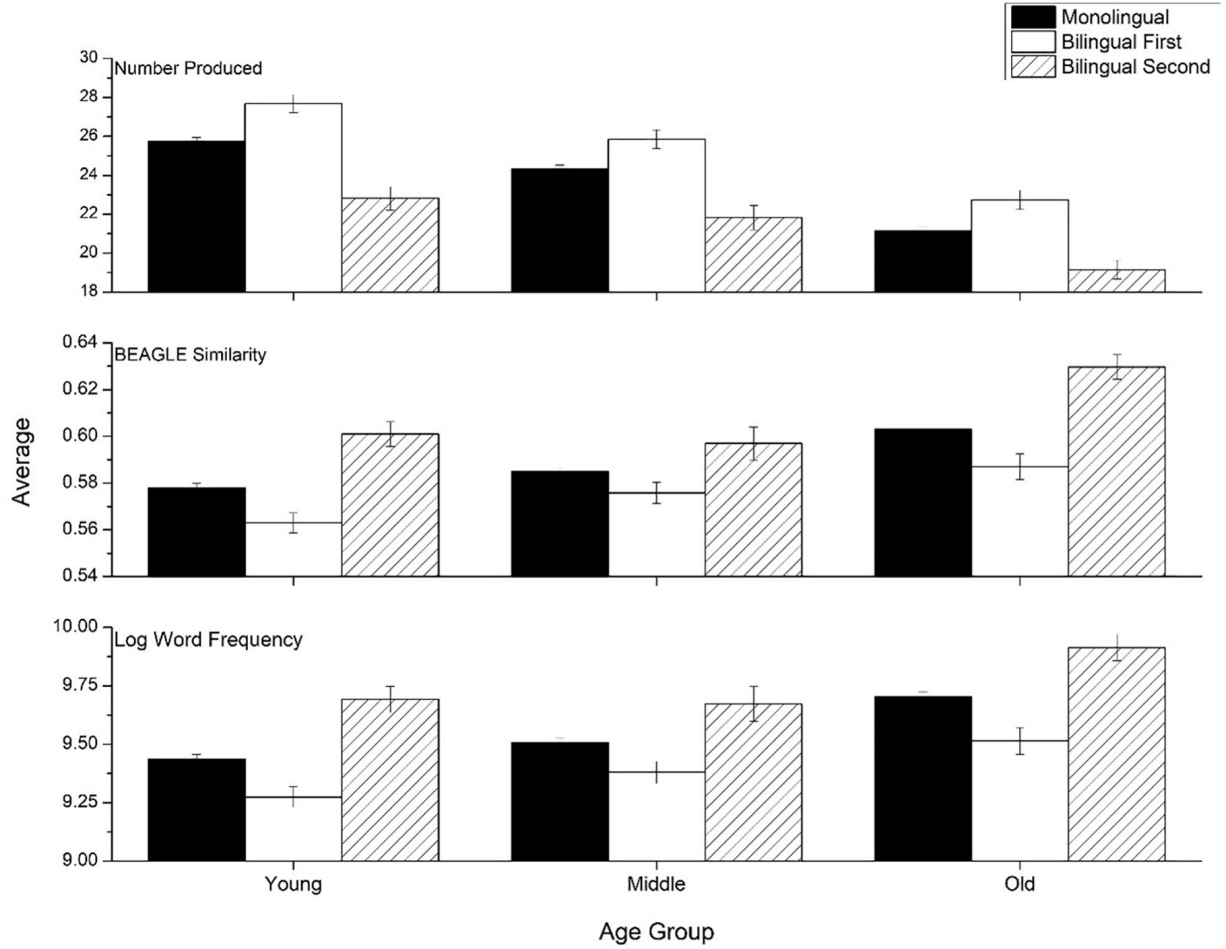
Means for the three psycholinguistic variables across the three language groups by age group. Error bars represent 95% confidence intervals.

Fig 3 shows performance in each age group, separated by language group. The language-group differences shown in Fig 1 are consistent across the age groups. We found significant main effects of language group for total items [F(2, 13,776) = 174.79, p<0.001, η^2^ = .025], word frequency [F(2, 13,776) = 144.41, p<0.001, η^2^ = .021], and semantic similarity [F(2, 13.776) = 107.95, p<0.001, η^2^ = .015]. There were also significant main effects of age on number of items produced [F(1, 13,776) = 291.8, p<0.001, η^2^ = .045], word frequency [F(1, 13,776) = 98.95, p<0.001, η^2^ = .014], and semantic similarity [F(1, 13,776) = 92.52, p<0.001, η^2^ = .013]. There was no significant language group by age interaction for number of items produced [F(4, 13,776) = 1.699, *n*.*s*.], nor word frequency [F(4, 13,776) = 2.296, *n*.*s*.]. There was a marginally significant interaction for semantic similarity [F(4, 13,766) = 2.92, p = 0.02, η^2^ = .001]; however, given the sample sizes involved in this analysis, a marginally significant result is unlikely to be meaningful. Bonferroni post-hoc tests confirmed that previously described impact of language status was observed for each age by language status split (p<0.001).

To assess the possible effect of education, the three language groups were split into lower (high school diploma or less) and higher (post-secondary degree) education groups. For monolinguals, this resulted in 6,044 participants in the lower education group and 4,412 participants in the higher education group. For the L1 English group, the respective sample sizes were 591 and 1,336, while for the L2 English group the respective sample sizes were 785 and 593. These splits show that the L1 English group had a much higher proportion of highly educated individuals.

**Fig 4:**
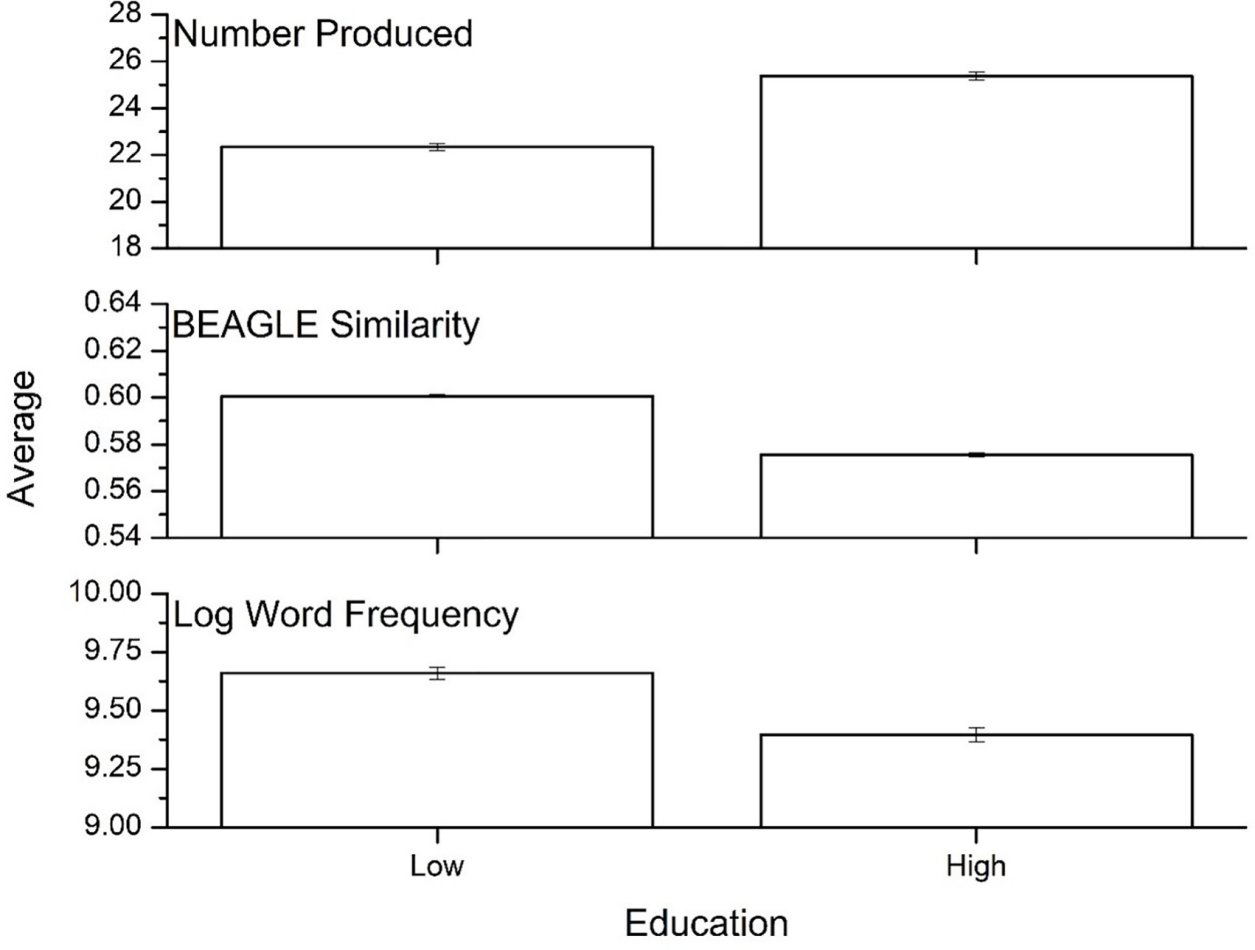
Means for the three psycholinguistic variables by education level. Error bars represent 95% confidence intervals.

Fig 4 shows the performance by education level, collapsed across the three language groups. There was a significant main effect of education on number of items produced [F(1, 13,776) = 757.31, p<0.001, η^2^ = .052; higher education group > lower education group], word frequency [F(1, 13,776) = 613.48, p<0.001, η^2^ = .049; lower education group > higher education group], and semantic similarity [F(1, 13,776) = 714.45, p<0.001, η^2^ = .043; lower education group > higher education group].

**Fig 5:**
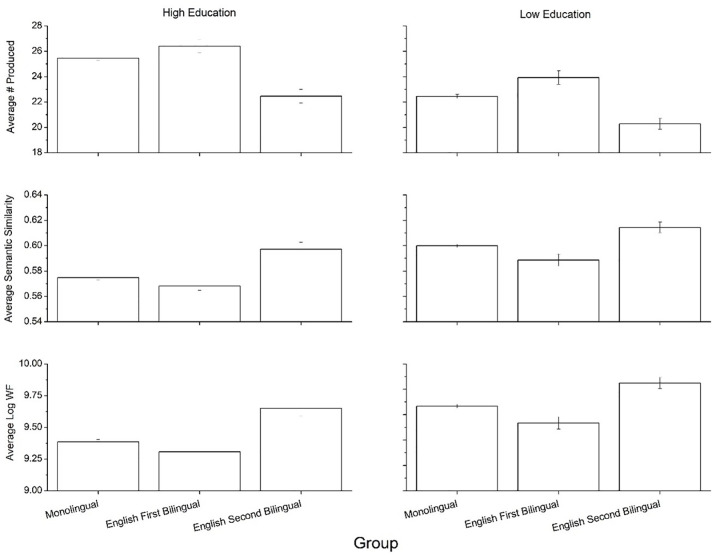
Means for the three psycholinguistic variables by language and education level. Error bars represent 95% confidence intervals.

Fig 5 shows performance by education level, separated by language status. It can be seen that effects of language group hold across different levels of educational attainment. We found significant main effects of language group on total items [F(2, 13,776) = 137.84, p<0.001, η^2^ = .02], word frequency [F(2, 13,776) = 114.68, p<0.001, η^2^ = .016], and semantic similarity [F(2, 13.776) = 84.75, p<0.001, η^2^ = .012], as well as significant main effects of education on number of items produced [F(1, 13,776) = 247.42, p<0.001, η^2^ = .018], word frequency [F(1, 13,776) = 233.13, p<0.001, η^2^ = .017], and semantic similarity [F(1, 13,776) = 196.44, p<0.001, η^2^ = .014]. Additionally, we observed marginally significant interactions between education and language group for number of items produced [F(2, 13,776) = 3.385, p = 0.03, η^2^ = .001], word frequency [F(2, 13,776) = 3.465, p = 0.03, η^2^ = .001], and semantic similarity [F(2, 13,776) = 3.314, p = 0.03, η^2^ = .001]. Again, given the sample sizes used here, marginal effects should be treated with caution. Bonferroni post-hoc tests confirmed that the L1 English group produced more items, of lower word frequency, and lower semantic similarity than the monolingual and L2 English groups within each education group (p<0.001). Thus, the superior verbal fluency performance of the L1 English bilinguals compared to monolinguals and L2 bilinguals holds across different education levels.

Finally, it is important to consider the possible impact of socio-economic status (operationalized here in five income categories). The five income categories are: 1) <$20,000, 2) $20,000-$50,000, 3) $50,000-$100,000, 4) $100,000-$150,000, and 5) >$150,000. Table 2 shows the sample sizes for the three language groups for these income levels. The overall sample was slightly reduced to 12,875 participants because not all participants disclosed their income. There is some variability in cell sizes here, with the L1 bilingual group having a greater proportion of high-income individuals compared to the other two groups.

**Table 2:** Sample sizes for the income levels and language groups.

Group	<$20k	$20k - $50k	$50k - $100k	$100k - $150k	>$150k
Monolingual	385	2042	3560	2005	1767
L1 Bilingual	52	256	544	477	507
L2 Bilingual	65	317	469	242	187

**Fig 6:**
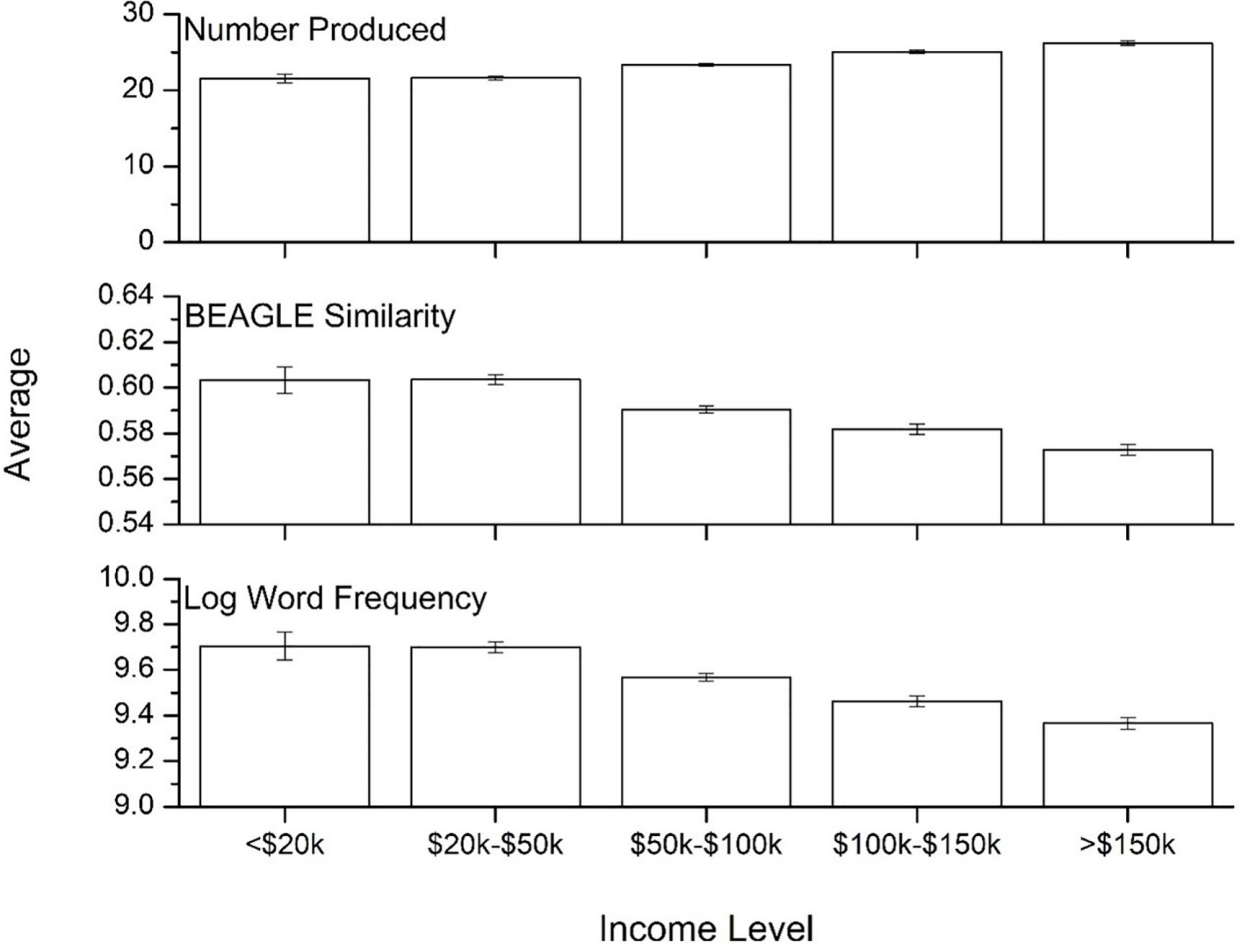
Means for the three psycholinguistic variables by income level. Error bars represent 95% confidence intervals.

Fig 6 shows the effect of language background on the three lexical variables across five income groups, collapsed across language groups. There was a significant effect of income on total items, such that individuals with higher incomes produced more items [F(4, 12,874) = 205.89, p<0.001, η^2^ = .06], and the words produced were of lower frequency [F(4, 12,874) = 117.19, p<0.001, η^2^ = .035] and semantic similarity [F(4, 12,874) = 103.2, p<0.001, η^2^ = .031], indicating that higher income is associated with better language performance. A Bonferroni post-hoc test found that there was no significant difference in any of the measures (p>0.1) for the <$20k and $20k-$50k income groups, but found that the higher income groups produced more items that were of lower frequency and less semantically similar (p<0.001). That is, the impact of income is similar to that reported above for age and education level.

**Fig 7:**
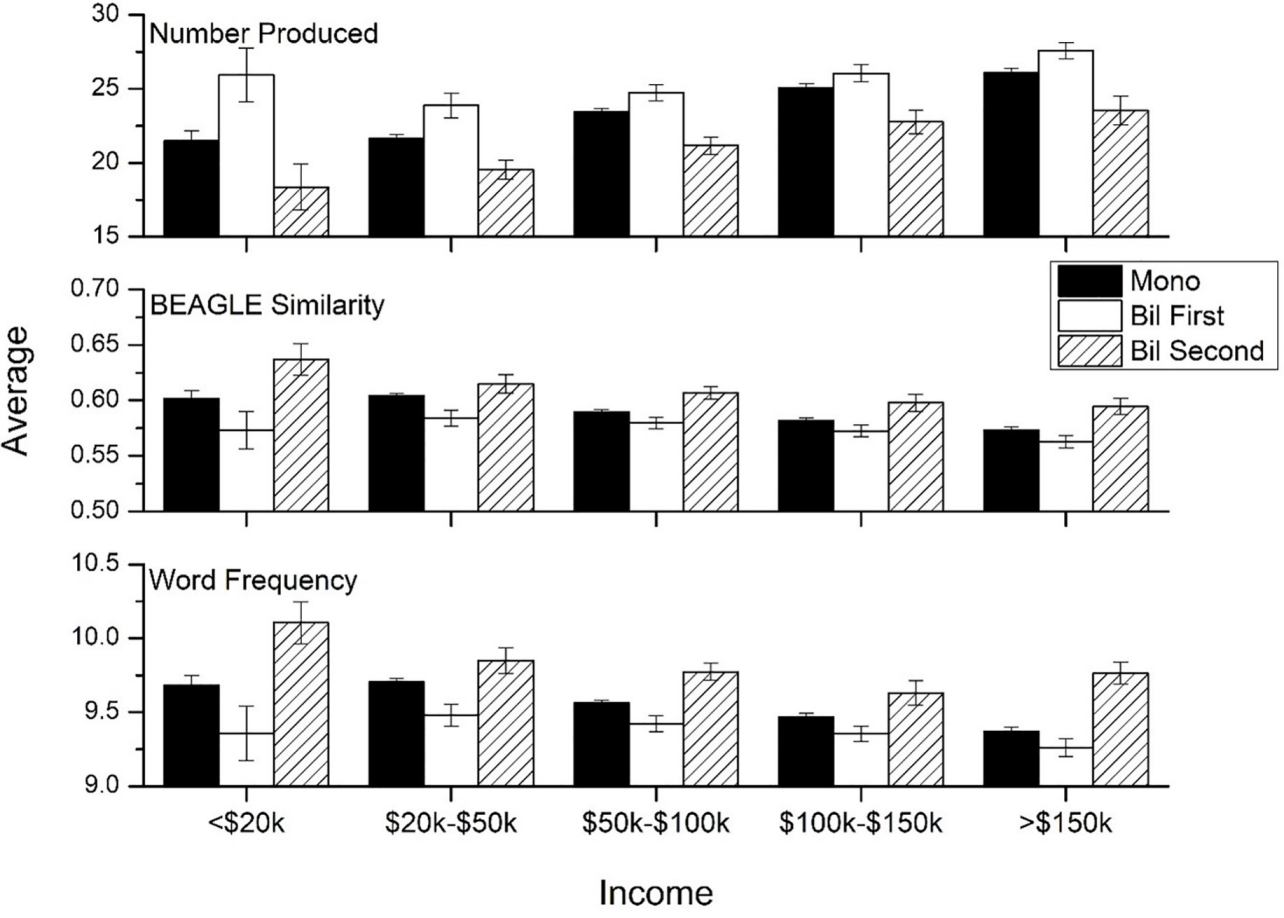
Means for the three psycholinguistic variables by language group and income level. Error bars represent 95% confidence intervals.

Fig 7 shows the results of income level when divided by language status. There were significant main effects of language group on total items [F(2, 12,874) = 108.79, p<0.001, η^2^ = .023], word frequency [F(2, 12,874) = 103.83, p<0.001, η^2^ = .016], and semantic similarity [F(2, 12,874) = 78.63, p<0.001, η^2^ = .012]. Likewise, we observed main effects of income level on number of items [F(1, 12,874) = 74.73, p<0.001, η^2^ = .023], word frequency [F(1, 12,874) = 37.66, p<0.001, η^2^ = .016], and semantic similarity [F(1, 12,874) = 32.49, p<0.001, η^2^ = .012]. Finally, there were marginally significant income by language group interaction effects for total items [F(2, 12,874) = 2.275, p = 0.02, η^2^ = .001], word frequency [F(2, 12,874) = 2.84, p = 0.004, η^2^ = .002], and semantic similarity [F(2, 12,874) = 2.32, p = 0.02, η^2^ = .001]. These findings indicate that there is little difference in the association between income and verbal fluency performance in the different language groups. Again, Bonferroni post-hoc tests confirmed that the L1 English group produced more items, had lower average word frequency, and lower average pairwise semantic similarity than the monolingual and L2 English groups within each income group (p<0.001).

## Discussion

The present study examined the effect of bilingualism on animal fluency performance in a large sample of middle-aged and older adults from the Canadian Longitudinal Study on Aging, using a common distributional model of semantics [the BEAGLE model; 27, 47]. To control for effects of completing the task in the second language, we included only participants who completed the task in English, and divided the bilingual group into those who spoke English as their first language (English L1 bilinguals) and those who spoke English as their second language (English L2 bilinguals).

We were interested in examining not just total items produced, but also characteristics of the items produced, which can provide a more fine-grained understanding of the impact of bilingualism on participants’ movement through semantic space. Specifically, we examined mean frequency of the items produced, as well as pairwise semantic similarity, a measure of how closely items are clustered together in space. We have previously reported increased production of higher frequency words that are more connected in semantic space in verbal fluency when task difficulty increases [21], in people who are developing a memory disorder [18], and in older adults relative to middle-aged adults [17]. That is, weaker semantic search abilities are reflected by production of items that are higher in frequency and more closely clustered together. Such a pattern in bilinguals would represent a bilingual *disadvantage*, while the opposite pattern (production of lower-frequency, lower pairwise similarity) would indicate a bilingual *advantage*.

Consistent with the latter possibility, we found that English L1 bilinguals produced more items in total than monolinguals, who in turn produced more items than English L2 bilinguals. L1 bilinguals also produced items that were lower in frequency and pairwise semantic similarity compared to monolinguals, while L2 bilinguals produced the most frequent and similar items. Additional analyses to investigate the effects of age, education, and income level found that older participants, those with lower education levels, and those with lower income levels produced fewer items, and the items they produced were higher in frequency and pairwise semantic similarity. That is, younger people, those with higher levels of education, and those in higher income groups all showed superior semantic search abilities. Crucially, however, the central finding of superior performance in L1 bilinguals compared to monolinguals, and in monolinguals compared to L2 bilinguals, was consistent across all age, education, and income groups.

These findings indicate that, at least in the present sample, L1 bilinguals are more skilled at semantic memory search than their monolingual and L2 bilingual counterparts. That is, they are better able to access lower frequency words and words that are further apart in semantic space, and consequently exhibit superior performance in the category fluency task. The lack of interaction between age and bilingualism indicates that these effects are not stronger with increasing age, contrary to previous reports suggesting a stronger bilingual advantage in older adults [for a discussion, see 37]. However, it should be noted that younger adults were not included in the present sample, and the possibility remains that age effects may be observed at earlier points in the lifespan.

The finding that L2 bilinguals exhibited the lowest performance is expected, given that these participants were performing the task in their non-dominant language. However, the finding of better performance in L1 English bilinguals compared to English monolinguals is more surprising. Previous research with smaller samples has found a bilingual disadvantage for semantic fluency tasks, even when completed in the dominant language [3, 31–33]. Moreover, in terms of the cognitive demands of the category fluency task, a bilingual advantage would not necessarily be expected; this task places demands on both language and executive function abilities, and previous literature has indicated that bilinguals exhibit robust disadvantages in language function compared to monolinguals [51], while the bilingual advantage in executive function is more equivocal [see e.g., 52].

However, the picture may be complicated by individual factors related to bilinguals’ language status and abilities. For example, Bialystok et al. [3] controlled for vocabulary size as a measure of proficiency, and found that high-proficiency bilinguals did not differ from monolinguals in category fluency performance, while both groups outperformed low-proficiency bilinguals. That is, high proficiency in the language of test administration eliminates bilinguals’ disadvantage in the category fluency task. Thus, one possibility is that the English L1 bilingual participants in our sample have higher English proficiency than the monolinguals. While proficiency data are not available for this group, participants in the CLSA were selected to be representative of the Canadian population, opening the possibility that higher English-language proficiency in bilinguals relative to monolinguals may be widespread and even typical within the Canadian anglophone population. While any explanation for this finding must remain speculative, one intriguing possibility is that higher language ability may contribute to the likelihood of acquiring a second language in people whose first language is the dominant language within their community, a hypothesis that could be further explored by comparing bilingual performance in English- and French-dominant regions in Canada. This possibility indicates the importance of considering underlying language ability when constructing participant samples in bilingualism research.

The relative importance of executive and semantic abilities in category fluency remains debated [see 10 for discussion]. As mentioned above, the participants in the present study were exclusively middle-aged and older adults, and it is therefore possible that better-preserved executive capacities in the bilingual sample may exert a stronger influence on task performance than would be observed in young adults, who are at the peak of their cognitive abilities. Indeed, it has been suggested that the bilingual advantage in executive function may be most observable later in life when cognitive functions have begun to decline [37]. Using a similar approach, albeit with a smaller sample, we previously reported that young adult monolinguals and bilinguals did not differ in fluency performance, consistent with this account [21].

From a clinical perspective, the present findings are also relevant. Category fluency is one of the most commonly-used tasks to measure language and executive function in clinical practice, and it is of paramount importance to develop a more refined understanding of the ways in which language background can influence performance. Previous research has suggested that low performance on fluency tasks in bilinguals is normal; however, the present findings contradict this assumption, and indeed indicate that bilinguals performing this task in their dominant language may perform *better* than matched monolinguals on this task. In the absence of reliable norms for monolinguals and bilinguals, the present result can contribute to clinicians’ judgment about whether poor fluency performance in a bilingual speaker is cause for concern.

The present study closely aligns with current trends in psycholinguistics, where the collection of large-scale data is becoming more commonplace (see for example, the English lexicon projects of Balota et al. [53]; and Mandera et al. [54]) [for a review, see 30]. The collection of big data sources of psycholinguistic data is complemented by the rise of distributional models of semantics, as they allow for word-level similarity values to be attained. In the current work, this allowed us to calculate items’ average pairwise similarity across groups, a standard information source used by theories of memory search [15, 16]. We have validated across multiple studies the finding that different groups use semantic similarity information differently [17, 20], suggesting that these models show promise in understanding the variability in performance of different participant groups.

There are several limitations to the present study. First, measurement of bilingualism is limited in the CLSA: participants are asked to state the language(s) in which they can hold a conversation, and which language they first learned at home. This information allowed us to categorize participants as functionally monolingual or bilingual, and to subcategorize bilinguals according to whether English was the first language, but more fine-grained information about age of acquisition and language history is not available. Second, while the present study controlled for age, education, and income, it was not possible to control for proficiency or vocabulary size. These factors exert an important influence on fluency performance and should be explored in depth in future research. Finally, it would be important to consider the potential impact of the specific languages spoken, as this factor likely has a strong influence on performance.

## Conclusion

The present study is the first to report on semantic search strategies in a large sample of monolingual and bilingual English speakers. We found that L1 English bilinguals outperformed monolinguals on a category fluency task, when the task was performed in English. Compared to monolinguals, bilinguals produced more total items, and these items were of lower frequency and pairwise semantic similarity. These findings indicate that, at least in the CLSA sample, bilinguals have superior semantic search capabilities in their first language compared to monolingual speakers of that language.
